# Editorial: Needs and challenges associated with the therapeutic use of novel natural products in weight control and diabetes management

**DOI:** 10.3389/fphar.2022.1107928

**Published:** 2023-01-16

**Authors:** Didem Deliorman Orhan, Esra Küpeli Akkol, Haroon Khan

**Affiliations:** ^1^ Department of Pharmacognosy, Faculty of Pharmacy, Gazi University, Ankara, Turkey; ^2^ Abdul Wali Khan University, Mardan, Pakistan

**Keywords:** diabetes, obesity, molecular docking, natural compound, weight controlv

We are honored to publish the Research Topic “*Needs and Challenges Associated with the Therapeutic Use of Novel Natural Products in Weight Control and Diabetes Management*” as guest editors. This thematic title aims to evaluate *in vitro* and *in vivo* studies, structure-activity relationships, and new therapeutic targets on isolated natural compounds for use in diabetes management and weight control. Four review/research articles of the scientific publications sent per the thematic title were deemed suitable for publication.

Jiang et al. summarize the potential therapeutic effects of artemisinin and its derivatives in Type 2 Diabetes Mellitus (T2DM), its mechanism, and associated complications, and provided a scientific reference for further detailed studies on this Research Topic in the future. Bai et al. investigate the effects and possible mechanisms of gastrodin, the main component of a traditional Chinese herbal medicine called *Gastrodia elata* Blume, in the treatment of T2DM. The results of the study showed that gastrodin promotes the phosphorylation of GATA1 *via* the PI3K/AKT pathway, increases the transcriptional activity of GATA1, and subsequently increases the expression level of USP4, thereby reducing the ubiquitination and degradation of insulin receptors, and ultimately improving insulin resistance. Yang et al. discuss the types and functions of histone methylation and its role in T2DM treatment. In addition, the role of histone methyltransferases and histone demethylases in the progression of T2DM is reviewed and potential applications of histone methylation modification as a treatment target are evaluated. Pekacar and Deliorman Orhan evaluate the *in vitro* antidiabetic, antiobesity, antioxidant, and antihyperlipidemic effect potential of *Pistacia atlantica* Desf. leaves with an activity-guided fractionation study. The presence of trigalloylglucose, digalloylglucose, methyl gallate, methyl digallate, 2″-*O*-galloyl-quercetin-3-*O*-hexoside, and myricetin-3-*O*-hexoside was determined by LC-QTOF-MS analysis in the sub-fractions that had the highest inhibitory effect on α-amylase and α-glucosidase enzymes among the sub-fractions obtained from the ethyl acetate extract of the leaves. In these published scientific articles, natural compounds that can be used in the treatment of T2DM, their mechanism of action, the effects of epigenetic modifications in T2DM, and potential applications of histone methylation modification as a treatment target are examined. Thank you to all the authors who contributed to this Research Topic of Frontiers in Pharmacology and we hope that it will be useful to its readers.

